# Periodontal Disease in Patients with Newly Diagnosed Systemic Lupus Erythematosus

**DOI:** 10.31138/mjr.20230724.pd

**Published:** 2023-07-24

**Authors:** Maryam Sahebari, Farzaneh Latifian Allaf, Nahid Nasrabadi, Ala Ghazi, Mandana Khodashahi, Fateme Absalan, Pegah Mosannen Mozafari, Shaghayegh Abbasi

**Affiliations:** 1 Rheumatic Diseases Research Centre, Mashhad University of Medical Sciences, Mashhad, Iran,; 2 Internal Medicine, Mashhad University of Medical Sciences, Mashhad, Iran,; 3 Dental Research Centre, School of Dentistry, Mashhad University of Medical Sciences, Mashhad, Iran,; 4 Oral and Maxillofacial Diseases Research Centre, Mashhad University of Medical Sciences, Mashhad, Iran,; 5 Private Practice Dentist, Sabzevar, Iran

**Keywords:** community periodontal index, periodontal disease, plaque index, systemic lupus erythematosus, Lupus, SLE

## Abstract

**Statement of the Problem:**

Most of the studies assessing the relationship between systemic lupus erythematosus (SLE) and periodontal disorder are focused on patients with previously diagnosed SLE whose periodontal hygiene may be influenced by immunosuppressive therapies.

**Purpose:**

This study aimed to evaluate the frequency of periodontal disease among patients with newly diagnosed lupus before starting immunosuppressive therapy and its association with presenting laboratory and clinical symptoms of lupus.

**Materials and Method:**

This case-control cross-sectional study was conducted on 36 consecutive newly diagnosed SLE patients before starting any treatment. The control group consisted of first-degree relatives of the patients whose demographic and social characteristics matched with the patients and who had no personal history of a disease. Periodontal indices included community periodontal index (CPI) and plaque index (PI).

**Results:**

Participants in both groups had some degree of periodontal disorder. The mean value of CPI was 1.47±0.82 and 1.31±0.72 in SLE patients and healthy subjects (P=0.84), respectively. Moreover, the mean values of PI were 1.15±0.55 and 1.17±0.46 in SLE patients and controls, (P=0.37), respectively. Besides, the frequency of periodontal disorders based on CPI score (positive: higher than two) was 22.2% in SLE patients and 16.7% in controls (P=0.55). Moreover, there was no association between periodontal disease and lupus-related clinical and laboratory characteristics in our patients.

**Conclusion:**

The frequency of periodontal disorders is similar between newly diagnosed lupus patients without undergoing immunomodulatory therapies and healthy controls with the same demographic and social characteristics. Moreover, periodontitis was not associated with clinical and laboratory symptoms of our patients.

## INTRODUCTION

Periodontal disease is a chronic inflammatory bacterial disease that affects 20–50% of the global population.^1^ It is one of the most common infectious oral diseases and is associated with inflammation of the tooth-supporting tissues, including the gums, periodontal ligament, cementum, and alveolar bone, which leads to chronic immune stimulation. This inflammatory process is caused by an imbalance in the interaction between the microorganisms of the dental biofilm and the overreaction of the immune systems.^2^

The innate or natural immune response induction depends on the severity and progression of periodontal disease.^3^ The response mechanisms involved in periodontal disease are very complex. During periodontitis, severe immune modulation occurs due to the effects of gram-negative bacterial lipopolysaccharides which induce lymphocytic immune damage in periodontal tissues. This group of diseases may appear more severely with malignant lesions, which led to systemic and mucosal immune responses in the pathophysiological processes of it.^4^

Systemic lupus erythematosus (SLE) is a multisystem and autoimmune disease with life-threatening manifestations, in which innate, cellular and humoral autoimmunity acts against various cell components.^5^ There are worldwide differences in the incidence and prevalence rates of SLE (0.3–241 per 100,000 people).^6^ It should be noted that the prevalence of the disorder is 40 per 100,000 in Iran.^7^ The SLE is characterized by increased activity of B and T immune cells, increased production of anti-nuclear antibodies (ANA), deposition of immune complexes in tissues, and ultimately tissue damage.^8^ The main symptoms of this disease in the oral cavity include oral ulcers and dry mouth. The most complications usually related to adverse drug reactions are candidiasis, gingivitis, and periodontal disease.^16,17^

High levels of activated B cells have been detected in both SLE and periodontal disorders with significant immune cell infiltration in target tissues. Simultaneously, very high levels of metalloproteinase and altered production of cytokines and C-reactive protein have been reported in association with SLE.^9,10^ Moreover, the periodontal disease also increases the serum concentrations of C-reactive protein, fibrinogen, and cytokines associated with atherosclerotic conditions.^9,11^ However, the association between periodontal disease and SLE is not still well understood. It is hypothesized that overexpression of B cells to antigen loads at the sites of periodontitis stimulates B cell activation involved in the SLE and leads to the formation of ANA.^9^

Infections can enhance the possibility of exacerbation of autoimmune diseases. Due to the high stimulatory effects of periodontal pathogen microorganisms, oral lesions associated with periodontal disease have increased expression of HSP60 which leads to stimulating the production of proinflammatory cytokines from tissue macrophages.^12^

There is also an association between periodontitis and inflammatory markers in patients with SLE and polyclonal B cell overreaction. Activation of B cells in SLE by periodontal bacteria may increase autoantibody production. It can explain data about the association between these antibodies and periodontitis in SLE.^9,13^

In practice, it has been reported that periodontal parameters in SLE have different levels of severity with conflicting results. Some studies have shown that the severity of periodontal parameters was similar in patients with SLE and the general population^5,14^; while other studies have indicated that the periodontal condition was worse in patients with SLE, compared to healthy individuals.^15,16^

Moreover, few studies are assessing periodontitis in newly diagnosed SLE patients. It is important since it could help to prevent periodontal diseases in patients with SLE. On the other hand, the role of immunosuppression induced by drugs in the occurrence of periodontitis will be discarded by the recruitment of newly diagnosed lupus patients.

## MATERIALS AND METHOD

This cross-sectional study with a control group was performed on patients with SLE at the Rheumatic Diseases Research Centre (RDRC) and Mashhad Dental Faculty both of which are affiliated to Mashhad University of Medical Sciences, Mashhad, Iran during 2018–19.

### Inclusion and exclusion criteria

The inclusion criterion for patients was newly diagnosed SLE patients based on Systemic Lupus International Collaborating Clinics (SLICC 2012), who were referred to Rheumatic Diseases Research Centre (RDRC). For controls, the age and sex-matched healthy relationships of those patients were asked about their health condition by interview and filling up a checklist. The exclusion criteria for both groups were the presence of underlying diseases affecting oral health, including diabetes, epilepsy, seizure, and other overlapping rheumatic diseases especially primary or secondary Sjogren syndrome; a history of smoking (cigarette or hookah), and drug addiction; and treatment history for lupus patients. Edentulous patients were excluded, also.

### Study design

Based on a previous study,^17^ the sample size was calculated at 36 subjects in each group considering α=0.05, β=0.1, and a power test of 90%. The participants consisted of patients who were newly diagnosed with SLE based on Systemic Lupus International Collaborating Clinics (SLICC 2012). Patients without treatment history were assessed for periodontal indices. The Control group included the first-degree relatives of the patients without a history of SLE who were matched with the patients in terms of demographic information, socio-economic status, age, and oral hygiene status.

Demographic information and the results of examinations and laboratory tests were collected and recorded in a checklist. Halitosis and dental hygiene habits were asked about in detail from participants.

Moreover, the periodontal examination was performed at Mashhad Dental Faculty, Mashhad University of Medical Silences. Periodontal examinations were performed by two periodontists to evaluate periodontal indices. The right upper and left lower quadrants of the jaws were selected as the sample.

Periodontal indices that were examined included community periodontal index (CPI) and Silness-Loe plaque index (PI). To examine the CPI index, the mouth is divided into sextants defined by tooth numbers: 18–14, 13–23, 24–28, 38–34, 33–43, and 44–48. A sextant should be examined only if there are two or more teeth present and not indicated for extraction. Next, the teeth were examined using the World Health Organization prop, and the code related to each tooth was recorded as follows: Code Zero: healthy, Code One: bleeding on probing, Code Two: the existence of calculus, caries, and overhang, Code Three: pocket depth is 4–5 mm and the black band of the probe can be seen, and Code Four: pocket depth is more than 6 mm and the black band of the probe cannot be seen. In summary, a special dental CPI-probe (WHO-probe) is used for the relevant examination. Periodontal diseases are classified into five degrees according to their severity ranging from 0 (healthy, inflammation-free gingiva and periodontium) to 4 (the most severe form of periodontitis with loss of function of the teeth). Periodontal diseases of degree 1 may be cured by improved domestic oral hygiene, and degrees 2 and 3 must be looked after and treated by a dentist. Degree 4 requires additional periodontal surgery, therefore degree 2 and more is considered established periodontitis.^2^

The PI examination was performed at three points for each tooth (scores were between 0 and 3). After that, the mean of the registered numbers of plate index was calculated and recorded:

- Zero: No plaque in the gingival area
- One: A layer of plaque on the free gingival margin adjacent to the tooth surface (the plaque may be detected by moving the probe against the tooth surface)
- Two: Medium density of soft derbies inside the gingival pocket on the gingival margin or near the tooth surface visible to the naked eye. Three: A large amount of soft debris inside the gingival pocket or on the gingival margin and adjacent to the tooth surface. As it was discussed, above the higher score for PI shows more severe periodontitis.^2^

### Statistical Analysis

Data were analysed in IBM SPSS software (version 23). Furthermore, descriptive indexes, including frequency (percentage) and mean were used to describe the quantitative and qualitative data. Moreover, the normality of data was evaluated using the Kolmogorov-Smirnov test. Eventually, the collected data were compared by t-test and its non-parametric equal Mann-Whitney U test. It should be mentioned that a p-value of less than 0.05 was considered statistically significant.

## ETHICAL CONSIDERATIONS

This research was approved by the Ethics Committee of Mashhad University of Medical Sciences (IR.MUMS. MEDICAL.REC.1397.694). The required data were collected and entered into a checklist via coding to maintain confidentiality. At the beginning of the study, the aims of the project were explained to the participants, and they were assured of the confidentiality of their information. Afterward, informed consent was obtained from all patients. Moreover, the participants were assured that they can leave the study at any time. Besides, all dental examinations were performed free of charge for the participants.

## RESULTS

The mean ages of participants in the case and control groups were 34.92 and 35.5 years respectively, (P=0.77). **Table 1** summarises the comparison between the two groups in terms of other demographic characteristics and general information. Based on Table 1, the two groups were matched in terms of gender, education, location of living, and oral health status including frequency of brushing and flossing. Brushing and flossing habits were asked in detail. There was no significant difference between the frequency of those habits between the two groups therefore, it was reported as a yes/no variable. However, the groups were different regarding bad breath (halitosis); accordingly, it was more prevalent in the lupus group in comparison to the control group (P=0.02). No patient and control subjects were smokers or addicts. **Table 2** tabulates the clinical and laboratory information of patients with SLE. The mean values of CPI were 1.47±0.82 and 1.31±0.72 in the case and control groups, respectively. No significant difference was observed between the two groups in terms of CPI (P=0.84). Moreover, the mean of PI was 1.15±0.55 and 1.17±0.46 in SLE patients and healthy control, respectively. The comparison between the two groups in terms of PI showed no significant difference between them (P=0.37).

**Table 1.** Comparison between lupus and control group in terms of other demographic and general information.

**Variables**		**Control**	**Lupus patients**	**p-value**
	**no (%)**	**no (%)**		
Gender	Female	34 (94)	29 (80)	0.07 *
	Male	2 (6)	7 (20)	
Education	Below high school	6 (17)	1)28(	0.52*
	High school	21 (58)	18(50)	
	Academic	9 (25)	8 (22)	
Location	Urban	34 (94)	33 (92)	0.99 *
	Rural	2 (6)	3 (8)	
Brushing	Yes	33 (92)	31 (86)	0.71 *
Flossing	Yes	14 (39)	12 (33)	0.62 *
Halitosis	Yes	8 (22)	17 (47)	0.02 *

* Chi-square test

**Table 2.** Clinical and laboratory information of patients with SLE.

**Variables**	**No**	**Percentage**		**No**	**Percentage**		**Mean±**	**SD**
Central nervous system disorder	3(8.3)		Decreased C4	13(36.1)		White blood counts /L	6998±3524	
Renal disorder	6(16.7)		Decreased C3	8(22.2)		Platelet count/L	244×10^3^±946×10^3^	
Cardiac disorder	3(8.3)		Positive anti-smooth muscle	9(25)		Hemoglobin g/L	12.1±1.6	
Arthritis	25(69.5)		Positive anti-SSB/La antibodies	4(11.1)		Creatinine (mg/dl)	0.95±1.24	
Serositis	12(33.3)		Positive anti–Scl-70 antibodies	0(0)		Anti-DNA ratio*	2±2.1	
Cutaneous disorder	26(72.2)		Positive anti-histone antibodies	4(11.1)		C3 ratio*	123±0.39	
Anemia	15(41.7)		Positive anti-SSA antibodies	12(33.3)		C4 ratio*	1.89±1	
24-hour urinary protein>500 ml/g	9(25)		Positive anti-Jo-1 antibodies	1(2.8)				
Positive Antinuclear Antibodies	35(97.2)		Positive anti-Ro52 antibodies	11(3.6)				
Positive anti-Antinuclear Antibodies	20(55.6)		Positive anti-nucleosome antibodies	8(22.2)				
Positive anti-RNP antibodies	2(5.6)							

Anti-DNA, C3 and C4 Ratio: the ratio of patient’s lab result/lab reference according the special laboratory detection kit.

**Figure 1.**
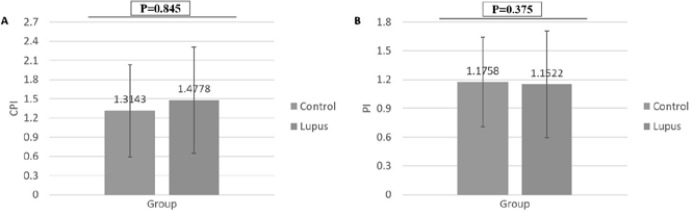
Comparison of community periodontal index (CPI) and plaque index (PI) between patients with lupus and healthy control group.

**Figure 1** shows the comparison of CPI and PI in patients with lupus, compared to the healthy controls.

The findings indicated that both patients and healthy individuals had some stages of periodontal disorder. The frequency of periodontal disorders based on CPI score (positive: higher than two) was 22.2% in patients with SLE and 16.7% in the healthy control group (P=0.55). We compared periodontal disorders based on the CPI score in lupus patients in terms of clinical and laboratory symptoms. Important lupus-related indices such as anti-dsDNA, C3, C4 values in para-clinic, and clinical disease manifestations including hematologic, musculoskeletal, kidney, cardiac, and central nervous system involvement compared in patients with and without periodontitis. No significant statistical difference was found between the detection of periodontitis in the patients with and without presenting clinical and abnormal laboratory manifestations (data not shown).

## DISCUSSION

Based on the obtained results, different stages of periodontitis were observed in both newly diagnosed lupus patients without previous treatments, and healthy sex, age, and cultured match healthy controls. Moreover, no difference was reported between the two groups in terms of PI and CPI (periodontal severity markers). No relationship was observed between periodontal disorders and clinical and laboratory manifestations of lupus. Several studies have been conducted to investigate the association between periodontal disorders and SLE; however, the results are conflicting.^16–19^ The present study is one of the few studies assessing periodontal conditions in patients with newly diagnosed SLE before starting glucocorticoids and immunosuppressive therapies.

A nationwide large observational cohort study in Taiwan was conducted on a large sample size (more than 7000 people) and its follow-up results confirmed a weak correlation between periodontitis and the development of SLE. The risk of lupus development in the population with periodontitis was 1.2 times more, compared to the others.^20^

A study performed by Wu et al. showed that the strongest relationship between periodontal disease and SLE was when the last periodontal disease-related visit was less than three months ago. They also found that smoking is a major risk factor for periodontal disease and SLE, while these variables were not assessed in the aforementioned study.^21^

Several case-control cross-sectional studies reported some correlations between periodontal indexes and lupus and some studies reported the opposite.^14–21^ This may be due to genetic differences between samples and other interfering variables especially smoking, addiction, behavioural hygiene, and overlapping with Sjogren syndrome in which dry mouth is an important bias.^14–17^

Based on the literature review, there are three systematic reviews and meta-analyses in this area. Rutter-Locher et al. reported that the risk of periodontitis in patients with SLE is 1.76 times more than in healthy individuals. However, they found no differences between SLE patients and healthy individuals in terms of other periodontal variables, such as probing depth and loss of clinical attachment level.^22^ The relationship between periodontitis and SLE was confirmed in a meta-analysis performed by Zhong et al. They reported a higher prevalence of bleeding on probing and clinical attachment loss in the SLE group in comparison to the reference group. However, they found that the patients with SLE and the reference group had no difference in terms of mean plaque index, gingival index, and pocket depth.^23^ Another systematic review indicates similar genetic vulnerability and environmental background as two main predisposing factors of SLE and periodontitis diseases. In this regard, the role of immune dysfunction and immunosuppressive treatment should be considered in the initiation and development of CP.^24^ Regarding what is related to lupus disease activity and periodontitis, some studies showed that the severity of lupus disease activity was related to poor oral hygiene and a high incidence of dental caries.^25,26^

Some co-variables, such as lifestyle, and social and economic status, have not been evaluated in most previous studies on this topic.^5,15–20,27^ Moreover, most of those studies were conducted on cases previously diagnosed with SLE who underwent corticosteroid therapy as part of their treatment process which predisposes patients to a higher risk of periodontal disease.^15,16,27,28^

Patients previously diagnosed with lupus gradually develop joint disorders and mood changes associated with a decrease in oral hygiene, such as a decrease in brushing frequency which, in turn, leads to an increase in periodontal disorders.^25^

## STRENGTHS AND LIMITATIONS

Only a few studies have assessed the relationship between periodontal disorder and lupus in patients newly diagnosed with SLE. The key strength of the present study is the use of family members of the patients as the control group, which eliminates the confounders of the socioeconomic status of periodontal disorders. On the other hand, newly diagnosed patients enrolled to investigate the role of periodontal disease in the development of native lupus with the elimination of immunosuppressive therapy and chronic illness-related mood disturbance on oral hygiene.

The main limitation of this study was the small sample size. This study was conducted only on women with SLE; therefore, the obtained results could not be generalised to other populations.

## CONCLUSION

This study indicates that the frequency of periodontal disorders between newly diagnosed SLE patients and healthy controls is similar. Besides, periodontitis had no relationship with clinical and laboratory symptoms in patients with systemic lupus erythematosus.
